# Using Latent Dirichlet Allocation Topic Modeling to Uncover Latent Research Topics and Trends in Renal Cell Carcinoma: Bibliometric Review

**DOI:** 10.2196/78797

**Published:** 2026-01-16

**Authors:** Javier De La Hoz-M, Karime Montes-Escobar, Carlos Alfredo Salas-Macias, Martha Fors, Santiago J Ballaz

**Affiliations:** 1 Universidad del Magdalena, Santa Marta, Colombia; 2 Departamento de Ciencias Agrícolas, Facultad de Ingeniería Agrícola, Universidad Técnica de Manabí, Portoviejo, Ecuador; 3 Departamento de Formación y Desarrollo Científico en Ingeniería, Facultad de Ingeniería, Ciencia y Tecnología, Universidad Bernardo O’Higgins, Santiago de Chile, Chile; 4 Laboratory of Agroecosystems Functioning and Climate Change – FAGROCLIM, Departamento de Ciencias Agronómicas, Facultad de Ingenierías Agroambientales, Universidad Técnica de Manabí, Santa Ana, Ecuador; 5 Facultad de Ciencias de la Salud, Escuela de Medicina, Universidad de Las Américas, Granados vía a Nayón sin número, Quito, 177022, Ecuador, 593 95016627; 6 Facultad de Ciencias de la Salud “Dr. Enrique Ortega Moreira”, Universidad Espíritu Santo, Samborondón, Ecuador

**Keywords:** bibliometrics, carcinoma renal cell, knowledge discovery, machine learning, research

## Abstract

**Background:**

Renal cell carcinoma (RCC) is a common, often lethal kidney cancer that originates in the renal cortex. Its incidence is rising, and major factors include smoking, obesity, and hypertension, though its etiology is uncertain. While surgery is effective for localized RCC, treatments for metastatic RCC have advanced significantly due to better diagnostic, prognostic, and predictive tools. Despite this progress, challenges remain, including long-term drug resistance and the complexity of RCC as a diverse group of diseases rather than a single entity.

**Objective:**

The aim of this bibliometric review was a comprehensive analysis of the topics and trends in RCC research, offering a foundation for future investigations.

**Methods:**

We used R “Bibliometrix” to conduct a bibliographic search in Scopus and PubMed covering publications from 1975 to 2023 to statistically assess the distribution of publications associated with RCC by year, journal, and country. Topic modeling of RCC research was conducted using latent Dirichlet allocation, a Bayesian network-based probabilistic algorithm that identifies unobserved thematic clusters in a collection of text documents. Trends in the retrieved themes were then characterized by using regression slopes over time, across countries, and in different journals. These trends were visualized as a heatmap, which was then used for hierarchical clustering to group similar topics based on their correlation strengths.

**Results:**

A total of 35,228 documents from 3070 sources were found, with a steady yearly growth of 9.86% and 118 participating countries. Thirty topics with the best coherence score were found in 8 crucial domains: treatment and therapies, biomolecular and genetic characteristics, disease characteristics and progression, diagnosis and evaluation, metastasis and dissemination, epidemiology and risk factors, related conditions, and pathological features. The pertinent clustergrams that resulted from the heatmaps mirrored the latent Dirichlet allocation’s algorithm identification of major RCC research subjects.

**Conclusions:**

Over 50 years, RCC research’s focus has shifted from diagnosis and assessment to a more thorough understanding of disease characteristics and progression. Because many patients are diagnosed with abdominal imaging studies, an emerging topic in RCC is diagnostic imaging and radiological evolution. The advances in omics technologies and the function of microRNA signature in the progression, diagnosis, therapy targeting, and prognosis of RCC have garnered a lot of attention. The discovery of the genetic background has enhanced our understanding of the growth of RCC. Drug resistance, local RCC ablation, and postoperative surveillance of RCC recurrence following nephrectomy are key future research avenues. The next generation of drug-targeted therapy and immunotherapy will make it possible to successfully treat metastatic RCC following nephrectomy. Neglected topics include the association between ferroptosis and RCC, the long-term assessment of novel treatments, and the application of artificial intelligence on RCC. Our bibliographic review delivered pertinent data for clinical decision-making and the planning of future RCC research.

## Introduction

Renal cell carcinoma (RCC) is the term coined to describe the malignant transformation of proximal renal tubular epithelium within the renal cortex [1]. RCC accounts for approximately 90% of all renal malignancies [2] and for approximately 2% of all cancer diagnoses and cancer deaths worldwide [3]. With a 2:1 ratio of new diagnoses, men are more likely than women to be affected by RCC, whose incidence rises significantly with age. RCC is a serious health problem since it is often a lethal kidney cancer with an increasing incidence worldwide. Its relevance is also tied to challenges in early diagnosis and the development of aggressive subtypes like those involving tumor thrombus. RCC’s etiology is uncertain. The 3 main risk factors for RCC include being overweight, having hypertension, and smoking cigarettes [3]. Medical disorders such as chronic kidney disease, hemodialysis, kidney transplantation, polycystic kidney disease, and renal stones are additional risk factors for RCC. Numerous dietary, occupational, environmental, and lifestyle factors have also been linked to the development of RCC [4]. Even though the majority of RCCs are sporadic, 3%‐5% of all RCC diagnoses occur in patients younger than 46 years, which suggests an underlying RCC form that is inherited [5].

In terms of treatment, surgery is a successful strategy for managing localized RCC, but conventional chemotherapy is ineffective for treating metastatic RCC. Thankfully, during the past 10 years, amazing progress has been made in treating metastatic RCC, resulting in a significant drop in the cancer’s death rates despite a continuous rise in the number of individuals receiving a diagnosis. The main factor for the improvement in RCC survival over the past few decades has been the wide diagnostic, prognostic, and predictive methodologies that are currently available [6,7]. Despite these advancements, long-term RCC drug resistance is still a problem [8]. While accelerating RCC cures is critically needed, research on RCC faces various obstacles. Prior to the discovery of the *VHL* gene, kidney cancer was regarded as a single disease [9]. In fact, kidney cancer is a multitude of diverse diseases, each with its own genetic makeup [10]. This has delayed the search for a cure by impeding reproducibility across studies and appropriate interpretation of the research [11].

RCC offers an intricate and challenging research landscape that hinders scientific progress. The goal of this study was to conduct a comprehensive and up-to-date summary of the topic structure, novel research avenues, study trends, and knowledge gaps in RCC research. Conventional bibliometric methods, scientific mappings, and network visualization studies fall short in offering this kind of text analysis [12], since they frequently necessitate manual categorization or extensive, subjective human intervention [13-16]. Instead, we sought a corpus of text-based data for research trends and topics by using a topic algorithm model called latent Dirichlet allocation (LDA) [17]. We intended for RCC research documents “to tell the story themselves” and for topics to emerge on their own, without human intervention, and only based on their statistical characteristics.

## Methods

### Search Strategy and Data Collection

This study was based on data obtained from PubMed [18] and Scopus [19] as of April 6, 2023. We chose Scopus over other databases because of its superior coverage in the health sciences field, accurate indexing, and “federated search interface” (ie, functionality), which enables us to query the content found across its sources using a common or standardized search form. The authoritative and comprehensive PubMed is the top choice for searching medical and health sciences literature. Medical Subject Headings (MeSH) terms were used for the PubMed search to improve comprehensiveness. Raw data were stored in *TXT* and *CSV* files, respectively. The R “Bibliometrix” tool of the R Statistical software [20] was used to clean data and integrate the 2 databases’ unique publications into a combined dataset (with and without assigned DOI) [18,19,21-23]. At this point, duplicate documents with an assigned DOI (1809 in total) were eliminated. The inclusion criteria for the outcome were all the research documents, written in English in peer-reviewed journals, that were published between 1974 (the earliest article we found) and 2023, and that dealt with RCC. Books, book chapters, gray literature, and reports were not included to avoid noise. The search strings, which used a Boolean computation, are indicated in Table 1. Only those articles with the term “renal cell carcinoma” in their title or abstract were selected. The leader and the other authors reviewed the complete list of all possible acceptable publications. Their reliability and value to the field were based on criteria like the journal’s impact factor, author affiliations, and citation count.

**Table 1.** Enhanced information retrieval for research on renal cell carcinoma.

Database	Search data	Search string	Results, n
PubMed	April 6, 2024	“renal cell carcinoma”[Title/Abstract] AND “english”[Language] AND “journal article”[Publication Type] AND 1974/01/01:2023/12/31[Date - Publication]	38,577
Scopus	April 6, 2024	TITLE-ABS ( “renal cell carcinoma” ) AND PUBYEAR>1973 AND PUBYEAR<2024 AND ( LIMIT-TO ( DOCTYPE , “ar” ) OR LIMIT-TO ( DOCTYPE , “re” ) ) AND ( LIMIT-TO ( SRCTYPE , “j” ) ) AND ( LIMIT-TO ( LANGUAGE , “English” ) )	40,479

### Bibliometrix Analysis

Our bibliometric review procedure adhered to best practice guidance published elsewhere [24] (Checklist 1). A preliminary descriptive analysis of the retrieved information was carried out using the R package bibliometrix [22]. This open-source application analyzes publication and citation metrics using mathematical and statistical methods to obtain a broad picture of the scientific output that was within the purview of the study. The questions answered were as follows: (Q1) What are the primary research topics in RCC? (Q2) How have RCC research questions changed over time? (Q3) How are these research topics distributed across countries and scientific journals? Three levels of analysis—countries, sources, and authors—were included at this stage to answer the above questions.

The annual growth rate of publications was calculated using the Bibliometrix package in R, which computes the compound annual growth rate (CAGR) using the following equation:


(1) $$\text{CAGR}={\left(V_{f}/V_{i}\right)}^{\frac{1}{n}}-1,$$

where *V*_f_ is the number of publications in the final year of the study period, *V*_i_ is the number of publications in the initial year of the study period, and *n* is the number of years between the initial and final year.

### Latent Dirichlet Allocation

The unsupervised machine learning algorithm LDA [17] was applied to identify topics. Considered an extension of the probabilistic latent semantic analysis, it has its roots in Bayesian models [17,25]. Topics in LDA are thought of as multinomial distributions of vocabulary terms, in which each word has a given probability of occurring in a topic. This leads to the prominence of words that are more frequently used in a topic, creating clusters that reflect specific underlying themes. LDA does not require prior knowledge of the topics or the way they are presented in the texts. Rather, topics merely flow from the statistical properties of the data and the model’s underlying assumptions. For thematic analysis, it was decided to use abstracts rather than entire texts because topics are more coherent and ranked higher in large document collections. Inaccurate or noisy terms have less impact on topic word distribution [26].

The LDA model was validated using the Cv metric, which is grounded in the distributional hypothesis stating that words with similar meanings tend to coexist in similar contexts [27]. In other words, Cv rates the semantic similarity of words within a topic (ie, topic interpretability). The Cv score was calculated by looking at word co-occurrence statistics in a reference corpus and their conformity to human-like semantic interpretation. The package textmineR was used for such analysis, which made it easier to determine the ideal number of topics (*k*) for the study. Because higher scores indicate better interpretability, a model with the highest coherence score among those in the study rank (from *k*=4 to 50) was selected to reach a balance between granularity and thematic clarity.

### Identifying Research Topics

The procedure for identifying topics through LDA was divided into 3 stages: (1) preprocessing, (2) construction of the LDA model, and (3) assigning labels to topics. LDAShiny [28], an open-source R package that uses Bayesian inference for LDA and machine learning algorithms to improve the analytical process, was selected for the first 2 stages.

#### Preprocessing

Converting all documents into a standardized format for ease of handling was the objective of the stage known as “Text refining” [29]. Initially, textual data consisted solely of character sets. To enhance topic coherence, each abstract underwent tokenization using bigrams, which are consecutive unigram combinations. This process involves converting text to lowercase and removing punctuation marks, dashes, brackets, numbers, spaces, and “stop words.” The list of stop words was extracted from standard libraries such as Natural Language ToolKit and Snowball and was modified to include unrelated terms unique to the medical and technical domains.

The preprocessed data result in the creation of a document-term matrix in which each document is represented as a vector containing an unordered collection of words. If the corpus contains a total of V words, each document becomes a V-dimensional vector, with the value of each element representing the frequency of the corresponding word in the document.

#### Construction of the LDA Model

LDA assumes that topics are shared by all documents in the collection, while subject proportions vary stochastically between documents, as they are randomly extracted from a Dirichlet distribution [30]. Establishing the expected number of topics was done a priori, making it a nontrivial task to choose the right number of topics (*k*) for a given collection of items. Since the optimal number of topics was unknown beforehand, we generated different models ranging from 4 to 40 topics. We ran 1000 iterations for Gibbs sampling [31] and utilized the default values of the LDAShiny package for Dirichlet parameters α and β. We used Cv as the topic coherence measure of the topics generated by LDA models [32].

#### Assigning Labels to Topics

The LDA model generates topics without semantic labels. Given that algorithmic analyses are not always able to fully capture the implicit meanings of human language, manual labeling is widely recognized as a normal practice in topic modeling [32]. The manual topic labeling involved a diverse team of 7 experts, including the authors and independent scholars with backgrounds in oncology research and bibliometric analysis. Team members were provided with 2 sources of information: the lists of most frequently occurring words (presumably) provided by the model and a sample of 3 document titles with their corresponding summaries classified by the algorithm. They were asked to independently validate and summarize the identified topics and investigate existing literature to identify research trends, gaps, and influential works. Discrepancies between team members were resolved through remote communications to minimize bias, improve rigor, and ensure the theme structures and semantic interpretation were aligned with the research objective. Reference [32] provides a guide to the procedures used to ensure the trustworthiness of the labels issued, with the difference that we used up to 16 annotators rating candidate labels.

By providing the most relevant and thematically aligned examples within each topic, these articles guaranteed readability and clarity. The 2 articles chosen allowed for a concise and efficient summary of each topic’s key ideas without overwhelming the analysis and the reader. This approach strikes a balance between realistic representation and practical interpretability. These articles were then condensed into succinct summaries that encapsulated the essence of each topic. This manual approach not only provided a gold-standard reference [32] but also ensured interpretability and utility in the context of the study.

### Quantitative Indices

For each topic, additional characteristics were revealed, especially at the journal and country levels, through statistical description based on the probability distributions of document-topic and topic-word acquired through LDA. To make results and findings more evident, we used certain quantitative indices suggested by Xiong and colleagues [33], which were obtained by adding document-topic and topic-word distributions. The indexes were described as follows.

The distribution of topics over time was obtained by the following equation:


(2) $$\theta_{k}^{y}=\sum_{m\in y}\theta_{mk}/n^{y},$$

where *m*ϵ*j* represents articles published each year, *θ*_*mk*_ is the proportion of the *k*th topic in each item, and *n*^*y*^ is the total number of articles published in the year.

Topic distribution across journals was defined as the ratio of the *k*th topic in the journal *j*: $\theta_{k}^{j}$ as indicated in the following equation:


(3) $$\theta_{k}^{j}=\sum_{m\in j}\theta_{mk}/n^{j},$$

where *m*ϵ*j* represents the articles in a particular journal, *θ*_*mk*_ is the proportion of the *k*th topic on each item, and *n*^*j*^ is the total number of articles published in the journal *j*.

Topic distribution across countries was defined as the ratio of the *k*th topic in the country *c*, as in the following equation:


(4) $$\theta_{k}^{c}=\sum_{m\in c}\theta_{mk}/n^{c},$$

where *m*ϵ*c* represents the articles in a particular country, *θ*_*mk*_ is the proportion of the *k*th topic on each item, and *n*^*c*^ is the total number of articles published in the country *c*.

### Statistics

With the purpose of facilitating the characterization of the topics in terms of their tendency, topic datasets were also submitted to simple regression slopes where the year, country, and journal were the dependent variables, while the proportion of the topics in the corresponding year, country, and journal was the response variable [34]. From the regression slopes, we determined the directionality of these trends and set a significance threshold of *P*<.01 (Eq2). Topics that showed statistically significant positive slopes were identified as having upward trends, while those with statistically significant negative slopes were in decline. Tendencies were finally visualized using the *ggcorrplot* library of R to represent correlation strengths as a heatmap matrix. The color-mapped matrix was subjected to advanced hierarchical clustering analysis in order to investigate and compile correlation datasets, given its visual form like a tree-shaped dendrogram. The *Agnes* function with *Ward’s* method showed the agglomerative hierarchical clustering of variables. Each leaf of the dendrogram corresponded to one observation (variable), and the fusion height showed the dissimilarity between 2 observations on the vertical axis. A cut height for cluster identification was calculated using the Average Silhouette method [35].

## Results

### Overview of the Dataset

**Figure 1.**
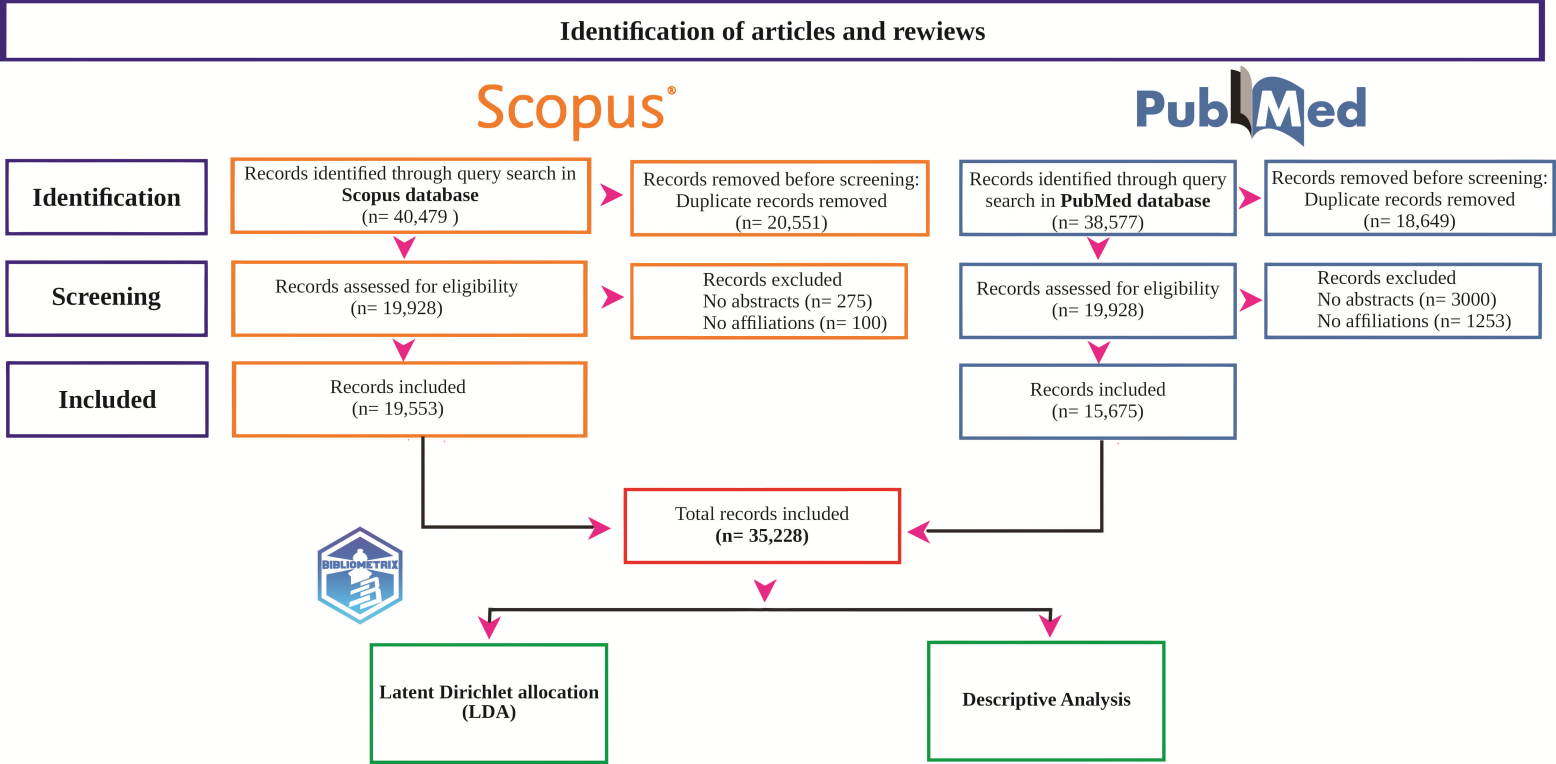
The workflow for article selection and bibliometric analysis in renal cell carcinoma using the PubMed and Scopus databases.

The consolidated dataset was obtained by combining the results and removing duplicates, totaling 39,856 articles (Figure 1). After the relevant Excel file was created, 4628 articles lacking titles, abstracts, or affiliations were removed. Within this extensive dataset, 35,228 documents were assembled, demonstrating an annual growth rate of 9.86%. The extensive summary of the key descriptive characteristics pertaining to RCC from 1974 to 2023 can be found in Table 2. A substantial number of information sources, 3070 in total, were revealed by the data. Given that the document’s average age was 11.6 years (the time since the publication of the examined articles), it is likely that much of the research was carried out some time ago. On average, however, each document received 32.35 citations, demonstrating their influence and recognition in the field (Table 2).

**Table 2.** Comprehensive overview of key descriptive characteristics and publication metrics on renal cell carcinoma from 1974 to 2023. Retrieved from the PubMed and Scopus databases.

Description	Results
Main information about data	
Timespan, range	1974‐2023
Sources (journals, books, etc), n	3070
Total documents, n	35,228
Annual growth rate (%), mean	9.86
Document age (y), mean	11.6
Citations per document, mean	32.35
Document contents, n	
Keywords plus	52,792
Author’s keywords	31,248
Authors, n	
Total authors	95,238
Authors of single-authored documents	608
Author collaboration	
Single-authored docs, n	769
Coauthors per doc, mean	7.55
Document types	
Original research	30,913
Review	4315

**Figure 2.**
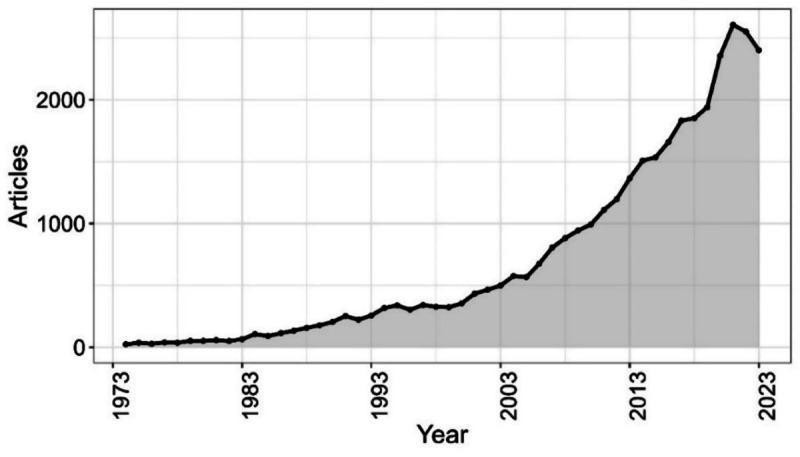
Annual production of documents on renal cell carcinoma from 1974 to 2023.

**Figure 3.**
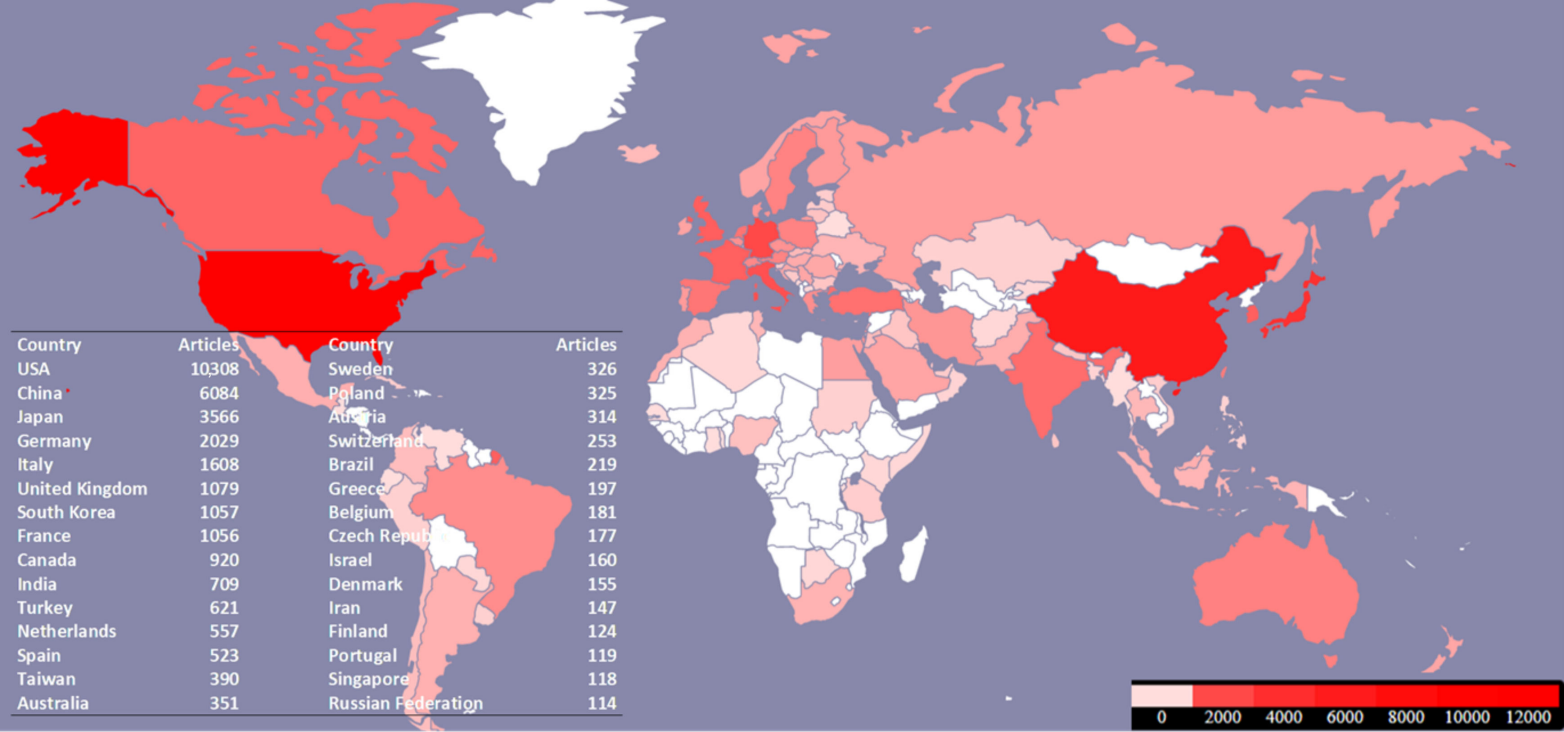
Distribution of geographical origins in the analysis of 35,228 published articles on renal cell carcinoma from 1974 to 2023. The table displays the top 30 countries with the highest research production.

With respect to the yearly output of documents on RCC, Figure 2 provides an overview of increased production from 1974 (24 articles) to 2023 (2401 articles). Throughout the 1980s and 1990s, the document production grew modestly. However, the 2000s saw a significant increase in production, reaching a peak in 2017 (1832 articles) and indicating a solid trend in recent years. Table 3 lists the top 30 scientific journals, while a global map (Figure 3) shows the 118 countries that were involved in RCC research. With 10,308 publications, the United States clearly was the top contributor.

**Table 3.** Top 30 scientific journals for research on renal cell carcinoma based on articles published between 1974 and 2023.

Source	Abbreviation	Articles, n
*Journal of Urology*	*J Urol*	1161
*Urology*	*Urology*	839
*Urologic Oncology: Seminars and Original Investigations*	*Urol Oncol*	560
*European Urology*	*Eur Urol*	542
*Frontiers in Oncology*	*Front Oncol*	481
*Cancer*	*Cancer*	478
*Clinical Genitourinary Cancer*	*Clin Genitourin Cancer*	474
*BJU International*	*BJU Int*	451
*International Journal of Urology*	*Int J Urol*	422
*Oncotarget*	*Oncotarget*	363
*PLOS One*	*PLoS One*	362
*Cancers*	*Cancers*	350
*Urologia Internationalis*	*Urol Int*	316
*International Journal of Cancer*	*Int J Cancer*	311
Clinical Cancer Research	*Clin Cancer Res*	308
*Oncology Letters*	*Oncol Lett*	307
*World Journal of Urology*	*World J Urol*	297
*British Journal of Cancer*	*Brit J Cancer*	291
*BMC Cancer*	*BMC Cancer*	273
*Scientific Reports*	*Sci Rep*	248
*American Journal of Surgical Pathology*	*Am J Surg Pathol*	236
*American Journal of Roentgenology*	*Am J Roentgenol*	210
*Journal of Clinical Oncology*	*J Clin Oncol*	206
*Cancer Research*	*Cancer Res*	200
*Human Pathology*	*Hum Pathol*	196
*Oncology Reports*	*Oncol Rep*	189
*International Urology and Nephrology*	*Int Urol Nephrol*	188
*International Journal of Molecular Sciences*	*Int J Mol Sci*	186
*Medicine (United States)*	*Med (United States)*	172
*Journal of Endourology*	*J Endourol*	165

### Latent Dirichlet Allocation

**Figure 4.**
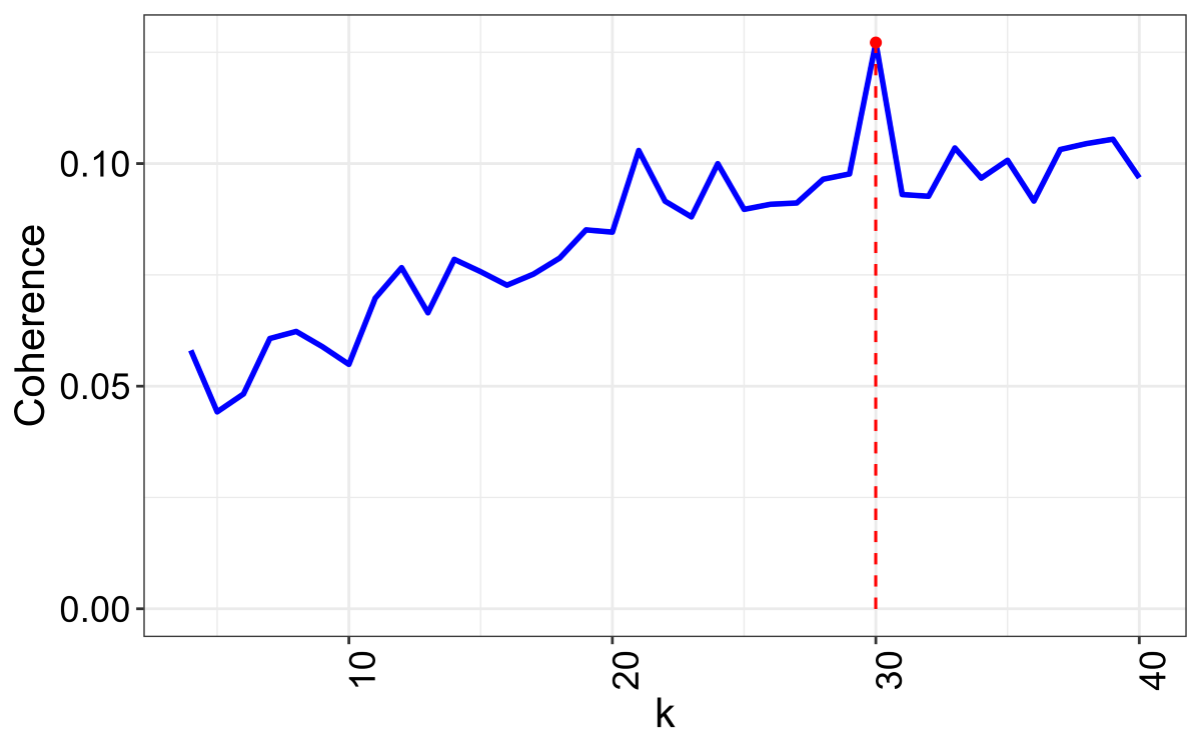
Evaluation of coherence scores for topic models in oncology across different numbers of topics (*k*). Coherence: measures the semantic quality of topics. Higher values indicate stronger word relationships. Topic models: identify thematic patterns in texts; in oncology, they reveal key research areas; *k* represents the number of topics in the model. Helps determine the optimal value for thematic analysis.

The methodological rigor, reproducibility, and accuracy of LDA were ensured using advanced bibliometric tools such as Bibliometrix and Textminer. There were 30 topics with the best coherence score in the LDA model (Figure 4). The terms with the highest probabilities and semantically relevant labels for each latent topic are shown in Table 4.

**Table 4.** Topics discovered from 35,228 articles on renal cell carcinoma published between 1974 and 2023.

Terms	Article numbers, n	Top terms	Label	Themes	Prevalence (%)
t_1	1411	inhibitor, sunitinib, treatment, target, drug, kinas, vegf, growth, factor, tki, sorafenib, resist, agent, tyrosin, growth_factor	Inhibitors in treatment	Treatment and therapies	3.455
t_2	563	rcc, level, patient, serum, control, rcc_patient, increas, group, concentr, elev, blood, distribut, plasma, healthi, compar	Serum levels and patient control	Diagnosis and evaluation	2.414
t_3	1240	express, tissu, normal, protein, tumor, level, correl, mrna, sampl, kidnei, posit, compar, marker, normal_tissu, express_level	Protein expression in tissues and tumors	Biomolecular and genetic characteristics	3.866
t_4	1370	gene, mutat, tumor, chromosom, genet, dna, loss, alter, famili, sequenc, region, identifi, methyl, variant, r	Genetic mutations and chromosomal alterations	Biomolecular and genetic characteristics	3.067
t_5	2113	rcc, clear, tumor, papillari, type, case, subtyp, carcinoma, featur, clear_rcc, prcc, posit, chromophob, histolog, pattern	Histological features and subtypes	Disease characteristics and progression	4.537
t_6	1293	tumor, surgic, thrombu, complic, oper, nephrectomi, surgeri, resect, laparoscop, blood, vena, ivc, postop, perform, approach	Surgical approaches and complications	Pathological features	3.053
t_7	939	vhl, Î±, hif, factor, Î², hif_Î±, protein, hypoxia, activ, induc, von, lindau, hippel, hippel_lindau, von_hippel	Hypoxia factors and related proteins	Pathological features	2.654
t_8	575	effect, treatment, ablat, control, local, search, evid, review, includ, meta, radiat, perform, systemat, outcom, percutan	Treatment effects and local ablation	Treatment and therapies	2.281
t_9	770	immun, pd, respons, immunotherapi, combin, nivolumab, checkpoint, ici, inhibitor, immun_checkpoint, therapi, checkpoint_inhibitor, treatment, death, anti	Immunotherapy and immune responses	Treatment and therapies	2.416
t_10	544	model, predict, score, risk, base, valid, group, curv, clinic, perform, cohort, featur, characterist, set, auc	Risk prediction models and clinical assessment	Epidemiology and risk factors	2.782
t_11	1593	imag, ct, enhanc, lesion, mass, contrast, evalu, tomographi, mri, comput, detect, phase, comput_tomographi, scan, find	Diagnostic imaging and radiological evaluation	Diagnosis and evaluation	3.633
t_12	1794	patient, month, surviv, median, mrcc, progress, o, group, pf, treat, metastat, free, line, progress_free, receiv	Survival and disease progression	Disease characteristics and progression	4.504
t_13	125	tumor, node, lymph, lymph_node, invas, metastasi, distant, involv, stage, crcc, posit, distant_metastasi, presenc, patient, node_metastasi	Metastasis and lymph node involvement	Metastasis and dissemination	1.776
t_14	724	metastat, metastas, metastasi, primari, patient, bone, site, rcc, lung, lesion, brain, primari_tumor, resect, pet, diseas	Metastasis and lesions in other organs	Metastasis and dissemination	2.724
t_15	324	patient, syndrom, develop, symptom, diseas, clinic, common, earli, relat, occur, sever, adult, hypertens, infect, manifest	Syndromes and clinical manifestations	Pathological features	2.108
t_16	1969	tumor, activ, human, effect, induc, increas, mice, antibodi, antigen, anti, deriv, specif, growth, line, cytotox	Tumor activity and immune responses	Disease characteristics and progression	4.218
t_17	2190	patient, nephrectomi, year, recurr, group, surgeri, follow, rate, rang, underw, local, radic, month, partial, diseas	Nephrectomy and recurrence	Metastasis and dissemination	5.242
t_18	1147	rcc, risk, ci, increas, associ, ag, patient, incid, compar, ratio, popul, year, mortal, interv, data	Risk factors and epidemiology	Epidemiology and risk factors	3.858
t_19	2049	patient, respons, treatment, dose, toxic, dai, week, event, advers, receiv, diseas, progress, evalu, phase, efficaci	Toxicity and adverse events in treatments	Pathological features	4.779
t_20	102	bladder, prostat, urolog, urinari, health, urotheli, tsc, prostat_cancer, malign, kluwer, lippincott, wolter, wolter_kluwer, william, wilkin	Urological cancers and related conditions	Related conditions	1.628
t_21	351	kidnei, diseas, long, term, transplant, function, long_term, develop, chronic, kidnei_diseas, egfr, diabet, dialysi, donor, recipi	Chronic kidney disease and transplant	Related conditions	1.967
t_22	1917	ccrcc, gene, clear, identifi, express, relat, prognosi, clear_ccrcc, ccrcc_patient, cancer, pathwai, biomark, potenti, data, genom	Gene expression and prognosis	Biomolecular and genetic characteristics	4.362
t_23	487	develop, molecular, potenti, recent, therapeut, provid, approach, clinic, understand, import, review, research, base, applic, strategi	Molecular advances and therapeutics	Pathological features	3.026
t_24	1388	surviv, patient, prognost, factor, independ, multivari, specif, cox, o, prognosi, free, prognost_factor, outcom, predictor, specif_surviv	Prognostic factors and survival	Disease characteristics and progression	4.137
t_25	202	cancer, type, lung, breast, kidnei_cancer, melanoma, cancer_patient, includ, lung_cancer, small, breast_cancer, research, acid, metabol, colorect	Nonrenal cancers and comparative analysis	Pathological features	2.354
t_26	555	malign, tumor, diagnosi, biopsi, benign, pancreat, neoplasm, thyroid, mass, case, diagnost, lesion, diagnos, carcinoma, small	Diagnosis and characterization of tumors	Diagnosis and evaluation	2.559
t_27	3022	rcc, mir, prolifer, inhibit, express, role, regul, assai, line, invas, effect, target, apoptosi, promot, migrat	Gene regulation and microRNA expression	Biomolecular and genetic characteristics	5.728
t_28	604	tumor, stage, grade, size, patholog, pt, tumor_size, low, nuclear, clinic, fuhrman, correl, necrosi, fuhrman_grade, tnm	Staging and pathological features	Pathological features	2.986
t_29	1168	therapi, treatment, clinic, target, improv, trial, advanc, system, review, manag, metastat, rcc, target_therapi, benefit, diseas	Advanced therapies and management of metastatic disease	Treatment and therapies	3.795
t_30	2699	case, report, year, present, rare, reveal, kidnei, left, mass, diagnosi, adren, report_case, examin, diagnos, literatur	Clinical presentation and diagnosis of rare cases	Diagnosis and evaluation	4.089

The 30 identified topics were in 8 crucial domains of RCC research:

- Treatment and Therapies: This category focused on different approaches to treating RCC, including targeted inhibitors (t_1), local ablation effects (t_8), immunotherapy responses (t_9), and advanced management of metastatic disease (t_29).
- Biomolecular and Genetic Characteristics: Here, the emphasis was on understanding the molecular and genetic makeup of RCC, covering topics like protein expression in tissues and tumors (t_3, t_22, and t_27) and genetic mutations and chromosomal alterations (t_4).
- Disease Characteristics and Progression: This category delved into the histological features and subtypes of RCC (t_5), as well as the dynamics of tumor activity and immune responses (t_16), and survival rates and disease progression (t_12 and t_24) and pathological features (t_28).
- Diagnosis and Evaluation: Topics in this category included diagnostic imaging and radiological evaluation (t_2 and t_11) for RCC detection and the characterization of tumors (t_26 and t_30) for accurate diagnosis.
- Metastasis and Dissemination: Here, the focus was on understanding how RCC spreads, including its involvement with lymph nodes (t_13), lesions in other organs (t_14), and the recurrence of the disease post nephrectomy (t_17).
- Epidemiology and Risk Factors: This category examined the risk prediction models and clinical assessment tools (t_10) used to evaluate RCC risk, as well as the epidemiological factors associated with the disease (t_18).
- Related Conditions: Topics here explored conditions related to RCC, such as urological cancers (t_20), chronic kidney disease, and transplant issues (t_21).
- Pathological Features: Finally, this category encompassed various pathological features of RCC, including surgical approaches and complications (t_6), hypoxia factors and related proteins (t_7), syndromes and clinical manifestations (t_15), toxicity and adverse events in treatments (t_19), molecular advances and therapeutics (t_23), and comparisons with nonrenal cancers (t_25).

### Topic Trends

**Figure 5.**
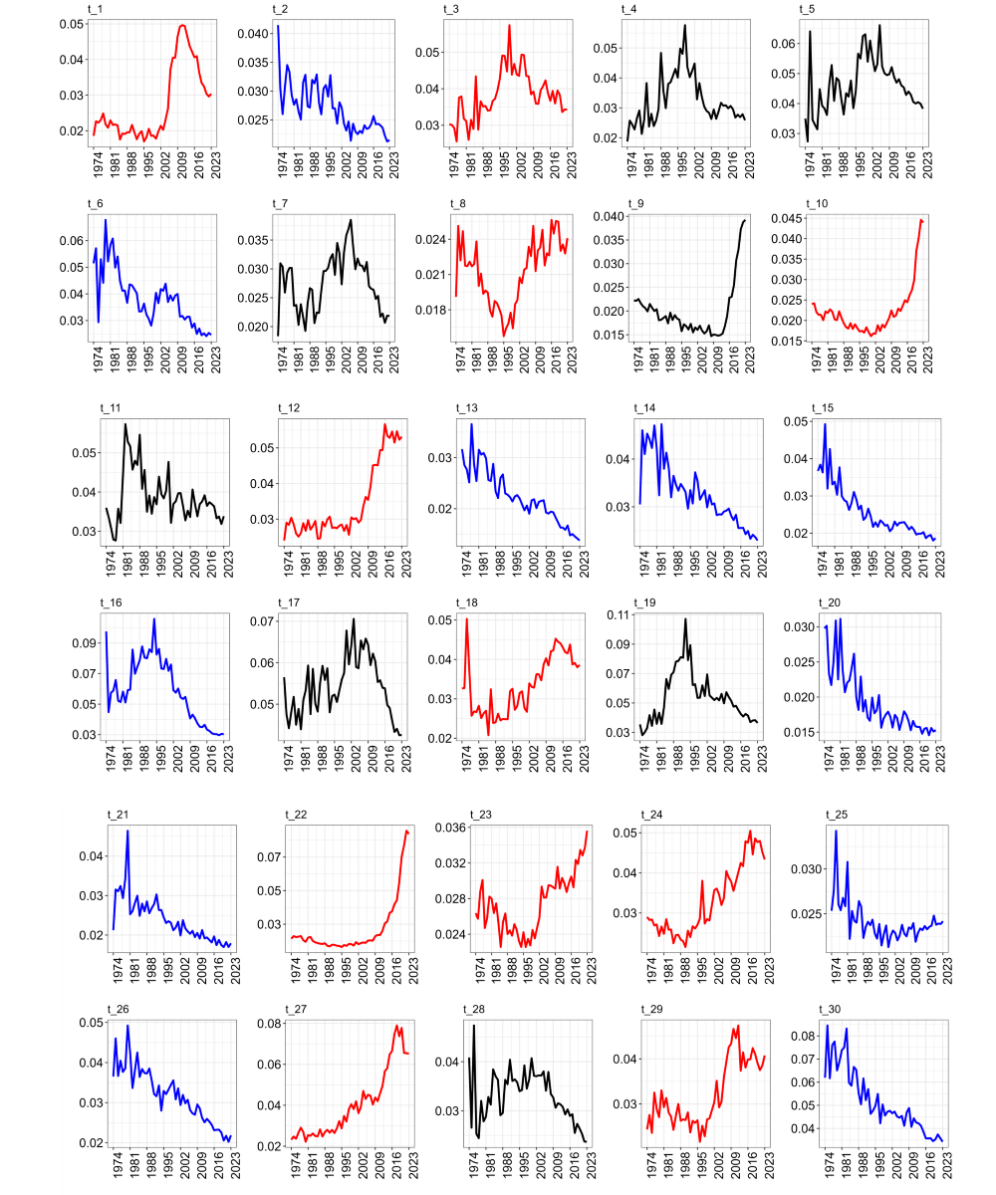
Trends of research topics in renal cell carcinoma between 1973 and 2023: increasing (red), decreasing (blue), and stable (black) topic dynamics over time.

The topic distribution by document $\theta_{m}$ was added to compute the average probability $\theta_{k}^{y}$ of all the articles published in a particular year to identify the trends (Figure 5). We found that the probabilities of some topics steadily increased over time (red). Black indicates topics with no discernible trend, whereas blue denotes topics with a decreasing behavior.

### Heatmaps

Although the discovery of 30 unique themes with high coherence scores, a granular and nuanced analysis of the research landscape, was made possible by the LDA method, there was a need for validating the theme output by a form of visual data analytics. Heatmaps helped examine and understand how thematic patterns were related to variables like publication year, country, and journal. Red highlighting indicates the strongest associations among variables, reflecting higher correlation levels.

**Figure 6.**
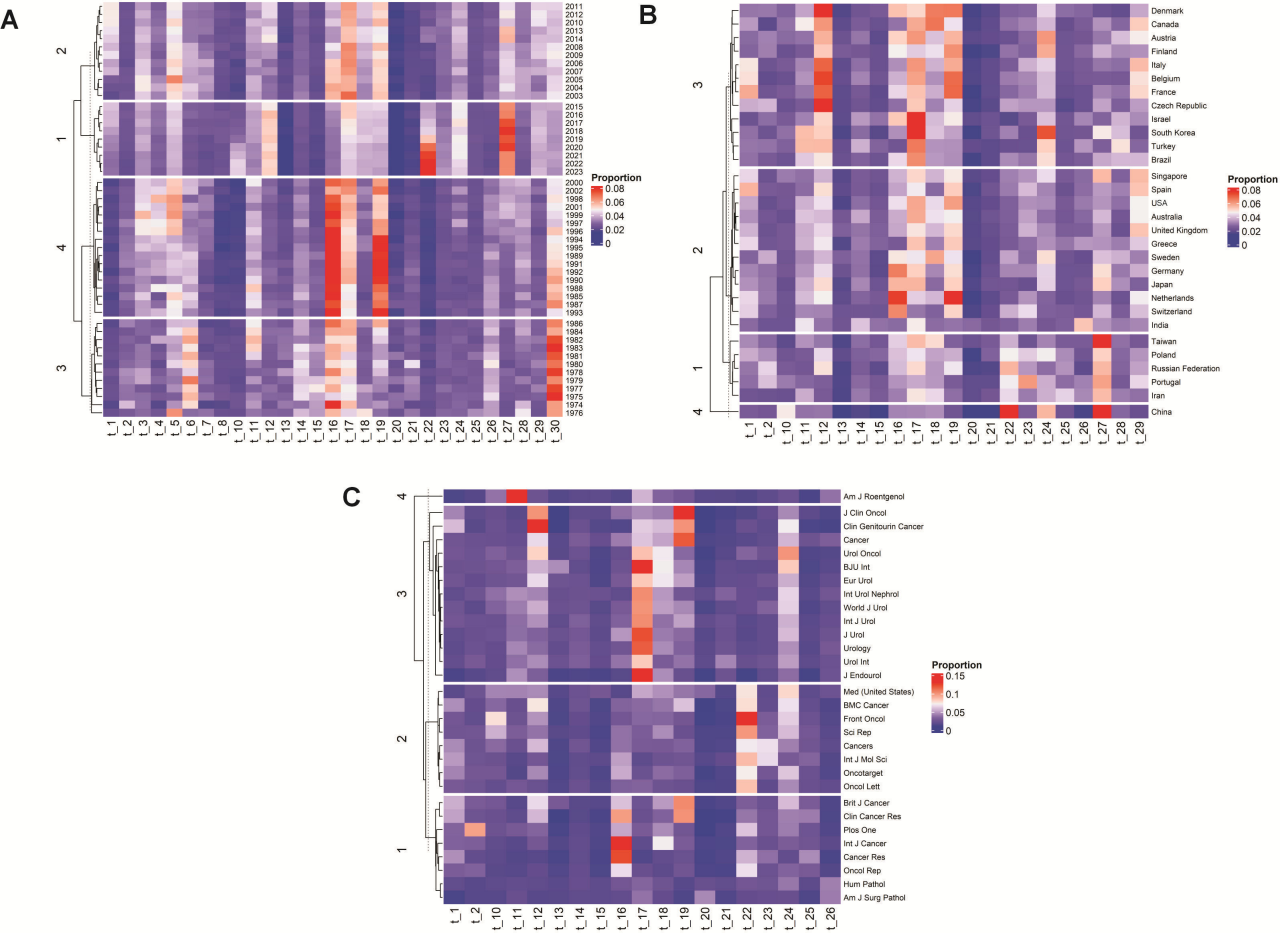
Heatmaps to correlate topics with year (A), country (B), and source (C).

Figure 6A illustrates correlations between specific topics and years. Red highlighting indicates the strongest associations among variables, reflecting higher correlation levels. For example, within Cluster 4, Topic 16 (t_16) “Tumor Activity and Immune Responses” is primarily associated with the years 1993, 1985, 1988, 1990, 1992, 1991, 1989, 1995, 1994, 1996, and 1999. Topic 17 (t_17) “Nephrectomy and Recurrence” exhibits stronger correlations with the years 1993, 1990, 1992, 1991, 1989, 1995, and 1994. In Cluster 3, Topic 22 (t_22) “Gene Expression and Prognosis” significantly correlates with the years 2020, 2021, 2022, and 2023, while Topic 27 (t_27), also addressing “Gene Regulation and microRNA Expression,” shows significant associations with the years 2017, 2018, 2019, and 2020. Finally, in group 3, Topic Thirty30 (t_30) “Clinical Presentation and Diagnosis of Rare Cases” is linked with the years 1982, 1983, 1981, 1978, 1977, and 1975.

Figure 6B presents the interactions between countries and topics across different groups. In group 1, Topic 27 (t_27), “Gene Regulation and microRNA Expression,” is predominantly correlated with Taiwan. Group 2’s topics, “Tumor Activity and Immune Responses” and “Toxicity and Adverse Events in Treatments,” are closely associated with the Netherlands. For group 3, “Survival and Disease Progression” shows significant correlations with the Czech Republic, Denmark, Belgium, France, and Italy, and “Nephrectomy and Recurrence” is notably linked with Israel and South Korea. Lastly, in group 4, “Gene Expression and Prognosis” and “Gene Regulation and microRNA Expression” are primarily connected with China.

Figure 6C outlines the dominant publication patterns for specific topics within various journals. In group 1, “Tumor Activity and Immune Responses” is predominantly linked with the *International Journal of Cancer* and *Cancer Research*. Group 2’s topic, “Gene Expression and Prognosis,” has a significant association with *Frontiers in Oncology*. In group 3, “Survival and Disease Progression” relates to *Clinical Genitourinary Cancer*, and “Nephrectomy and Recurrence” is associated with *BJU International*, *Journal of Endourology*, and *Urology*. Lastly, in group 4, “Diagnostic Imaging and Radiological Evaluation” is prominently linked to the *American Journal of Roentgenology*. LDA algorithm’s identification of the key RCC research themes was mirrored in the relevant clustergrams that emerged from the hierarchical cluster analysis of the heatmaps.

## Discussion

### Principal Findings

By displaying each topic as a group of related words, LDA was able to identify latent (hidden) topics within a corpus of documents and demonstrate how each document may be represented as a combination of these topics. According to this method, RCC research has evolved over the past 50 years from concentrating on surgery to comprehending its genetic underpinnings and the impact of new treatments like immune checkpoint inhibitors and targeted therapies, which have improved the prognosis of metastatic RCC.

The disease characterization, progression, and pathological features have dominated the RCC research landscape for the past 50 years (t_16 and t_19) [10]. Kidney cancer was regarded as a single disease until the *VHL* gene was discovered [9]. Since then, scientists have realized that kidney cancer is a multitude of diverse diseases, each with its own genetic makeup. Although the characterization of genetic mutations and chromosomal alterations linked to the growth of RCC [36,37] has recently improved our knowledge of this kidney cancer (t_22), RCC research does emphasize the necessity of assessment of treatment effects’ safety and efficacy, as well as surveillance of RCC recurrence following nephrectomy. Therefore, a better understanding of resistance mechanisms, molecular prognosis (t_22), and immunological responses (t_16) is essential.

The advances in omics technologies over the last 10 years constitute a promising area of personalized RCC cure [1,38,39]. In addition, the microRNA signature in RCC and its function in progression, diagnosis, therapy targeting, and prognosis of RCC (t_27) have also received particular attention [40,41]. Recent RCC research also moves toward earlier cancer detection through broad imaging and radiological evolution (t-11). RCC has a difficult pathological classification, since the histological analysis reveals three most recurrent sporadic types: clear-cell RCC (70%‐75%), papillary RCC (10%‐15%), and chromophobe RCC (5%) [42]. It is predicated on morphologies, architecture, underlying genetic abnormalities, and tumoral protein expression [43,44]. Pathologists can detect these malignancies more accurately and provide better treatment plans and patient outcomes if they are aware of the significance of these markers (t_12 and t_19).

A major cause for worry is the postoperative surveillance of RCC recurrence following nephrectomy (t_17) [45]. Research on local RCC ablation and assessment of treatment effects on safety and efficacy is still ongoing (t_12, t_16, and t_19). The discovery of tailored medication like tyrosine kinase inhibitors (also called TKIs), such as Sunitinib and Sorafenib, has been beneficial in treating metastatic RCC [46,47]. Another well-established component of RCC treatment is checkpoint inhibitor immunotherapy [48], which shows its superior therapeutic efficacy when combined with TKIs [49]. The next generation of TKIs and immunotherapy (t_16) [50] is being developed to overcome some unfavorable outcomes with kinase inhibitors [51,52] and immunotherapy (t_19) [53], as well as the emergence of drug resistance [54]. Only a small number of themes still earn little attention. The topics “Serum Levels and Patient Control” (t_2), “Hypoxia Factors and Related Proteins” (t_7), and “Risk Prediction Models and Clinical Assessment” (t_18) are overlooked and should require additional attention.

In line with the LDA analysis and our own scientific expectations and goals, it was also feasible to identify untapped topics that have not yet been covered by scholarly literature. Intriguing lines of inquiry are the prognostic value of vascular endothelial growth factor [55], endostatin [56], C-reactive protein [57], the hypoxia-induced pathway [58], and ferroptosis [59]. The involvement of chronic inflammation [60] and gut and urinary microbiota in immune modulation of metastatic RCC [61] remains poorly investigated. Clinical judgments and patient stratification in RCC may be enhanced by the creation of new predictive models [62] based on, for instance, genetic biomarkers [63]. The application of artificial intelligence is another potential topic that could help physicians in identifying RCC subtypes by analyzing computed tomography scans, as well as in deconstructing complex epidemiological and environmental factors that influence RCC occurrence, like hypoxia [64].

In contrast to traditional bibliometric analysis, the LDA approach effectively extracted potential themes and inferred implicit information from a large collection of documents. The LDA approach and other topic modeling methods like co-citation and keyword co-occurrence cannot be compared with the same conceptual granularity or depth. LDA goes beyond who cites whom (ie, intellectual connection and research lineage) and uncovers the underlying conceptual themes that bind the literature, which may not be immediately apparent from citation patterns alone. Unlike a list of keywords, which reveals basic relationships, LDA organizes these co-occurring words into meaningful higher-order themes, offering a more detailed knowledge of topic relationships and structure. For this reason, the LDA analysis was not affected by the potential simplicity of the keywords chosen in the study.

The subjectivity involved in manually labeling LDA topics, the possibility of missing publications by using only 2 databases, the linguistic bias introduced by excluding articles written in languages other than English, the constraints of the “bag-of-words” model which disregards grammar and context, and the effects of excluding literature like book chapters are some limitations of the study. Despite the constraints, LDA can disclose “unknown unknowns” by revealing unarticulated or unacknowledged themes. It can also give an overview of the research landscape to identify new topics and interdisciplinary connections, as well as demonstrate how old themes are resurfacing in new ones. As a result, LDA remains the most often used natural language model [13,14,65].

### Conclusions

This review offered a thorough summary of how research on RCC has changed over the previous 50 years. LDA helped identify important emerging trends in treatment development to address drug resistance and undesirable side effects, surgical techniques, and immunotherapy advancements, among other topics pertinent to clinical practice and medical research. In summary, this study presents a methodological synthesis of the development of RCC research and delivers pertinent data for clinical decision-making, early identification, and the planning of new biomedical research.

## Supplementary material

10.2196/78797 Checklist 1 Bibliometric analysis checklist.

## Abbreviations

LDA: latent Dirichlet allocation
MeSH: Medical Subject Headings
RCC: renal cell carcinoma
TKI: tyrosine kinase inhibitor
