# Supplementary material for: Using Latent Dirichlet Allocation Topic Modeling to Uncover Latent Research Topics and Trends in Renal Cell Carcinoma: Bibliometric Review
Source: JMIR Cancer. 2026 Jan 16;12:e78797. doi: 10.2196/78797 (PMC12810951; doi:10.2196/78797)
Supplement: Checklist 1 [file cancer-v12-e78797-s001.pdf]

SUPPLEMENTARY MATERIAL 1

Bibliometric analysis checklist

No.	Step	Practice questions	Yes/No?
Step 1	Aims and scope of the bibliometric study	Are the aims and scopes of the study well defined?	Yes
		Is the scope of the study large enough to warrant the use of bibliometric analysis?	Yes
Step 2	Selection of the techniques for bibliometric analysis	Do the chosen bibliometric analysis techniques meet the aims and scope of the study?	Yes
Step 3	Data collection for bibliometric analysis	Do the search terms exemplify the scope of the study?	Yes
		Is the coverage of the database adequate for the study?	Yes
		Is the data free of errors such as duplicates and erroneous entries?	Yes
		Does the final dataset fulfil the requirements of the bibliometric analysis techniques chosen for the study?	Yes
Step 4	Run the bibliometric analysis and report the findings	Can the bibliometric summary be easily understood by readers?	Yes
		Does the writing align with the bibliometric summary presented?	Yes
		Does the writing explain the peculiarities and implications of the bibliometric summary?	Yes
		Does the writing align with the target outlet for publication?	Yes
